# Reducing complication rates for repeat craniotomies in glioma patients: a single-surgeon experience and comparison with the literature

**DOI:** 10.1007/s00701-021-05067-9

**Published:** 2021-12-30

**Authors:** Ramin A. Morshed, Jacob S. Young, Andrew J. Gogos, Alexander F. Haddad, James T. McMahon, Annette M. Molinaro, Vivek Sudhakar, Nadeem Al-Adli, Shawn L. Hervey-Jumper, Mitchel S. Berger

**Affiliations:** 1 Department of Neurological Surgery, University of California, San Francisco, 505 Parnassus Ave., Rm. M-779, San Francisco, CA 94143-0112 USA; 2 School of Medicine, University of California, San Francisco, San Francisco, CA USA; 3 School of Medicine, Emory University, Atlanta, GA USA; 4 School of Medicine, University of Pittsburgh, Pittsburgh, PA USA; 5 School of Medicine, TCU and UNTHSC, Fort Worth, TX USA

**Keywords:** Complications, Glioma, Recurrence, Surgical resection

## Abstract

**Background:**

There is a concern that glioma patients undergoing repeat craniotomies are more prone to complications. The study’s goal was to assess if the complication profiles for initial and repeat craniotomies were similar, to determine predictors of complications, and to compare results with those in the literature.

**Methods:**

A retrospective study was conducted of glioma patients (WHO grade II–IV) who underwent either an initial or repeat craniotomy performed by the senior author from 2012 until 2019. Complications were recorded by discharge, 30 days, and 90 days postoperatively. New neurologic deficits were recorded by 90 days postoperatively. Multivariate regression was performed to identify factors associated with complications. A meta-analysis was performed to identify rates of complications based on number of prior craniotomies.

**Results:**

Within the cohort of 714 patients, 400 (56%) had no prior craniotomies, 218 (30.5%) had undergone 1 prior craniotomy, and 96 (13.5%) had undergone ≥ 2 prior craniotomies. There were 27 surgical and 10 medical complications in 30 patients (4.2%) and 19 reoperations for complications in 19 patients (2.7%) with no deaths by 90 days. Complications, reoperation rates, and new neurologic deficits did not differ based on number of prior craniotomies. On multivariate analysis, older age (OR1.5, 95%CI 1.0–2.2) and significant leukocytosis due to steroid use (OR12.6, 95%CI 2.5–62.9) were predictors of complications. Complication rates in the cohort were lower than rates reported in the literature.

**Conclusion:**

Contrary to prior reports in the literature, repeat craniotomies can be as safe as initial operations if surgeons implement best practices.

**Supplementary Information:**

The online version contains supplementary material available at 10.1007/s00701-021-05067-9.

## Introduction

Greater extent of resection has been shown to improved outcomes for patients with low and high grade gliomas both at initial presentation and recurrence [5, 11, 28, 31, 38]. However, some reports suggest that glioma patients undergoing reoperation may be more prone to certain complications [25]. The decision to reoperate is often a challenging one, and the potential benefits of a repeat surgical resection must be weighed against the risks of additional surgery as well as the potential benefits of other therapies such as radiotherapy or chemotherapy.

There are several concerns for why reoperation may pose an increased risk to patients. Prior anatomical landmarks may be obscured due to tumor recurrence and gliosis, and adhesions may predispose patients to cortical injury. Additionally, vascular supply to the skin, prior radiation, steroid use, and scar tissue may increase the risk for wound healing complications. Prior series including patients undergoing repeat craniotomy for glioma resection have reported complication rates between 5.7 and 48% [3, 6, 7, 11, 13, 15, 20, 22, 29, 31, 33, 37, 41, 43, 44].

Surgical experience has been shown to significantly reduce complications in other neurosurgical areas, and therefore, we wanted to determine whether complication rates for repeat craniotomies would be similar to rates for initial resections when surgery was performed by an experienced neurosurgical oncologist. The goals of the study were to assess the complication profile based on number of prior craniotomies, to compare results with those in the literature, and to discuss techniques for repeat craniotomies.

## Methods

### Inclusion criteria

After obtaining approval from the institutional review board (Study Number 15–17,500), the institutional tumor registry was searched for patients who underwent a craniotomy for glioma between 2012 and 2019, corresponding to modern documentation of complications in the electronic medical record at our institution. Consent was not required due to minimal risk posed to patients. Inclusion criteria for the cohort included age ≥ 17, initial or repeat craniotomy, a diagnosis of WHO grade II–IV glioma, operation performed by the senior author, and follow-up of 90 days unless death occurred prior (*n* = 0). Patient, tumor, and outcome data were collected retrospectively from the electronic medical record.

### Patient selection for repeat craniotomies

Repeat surgical intervention for recurrent glioma should be judiciously offered to patients based on each unique patient’s needs, baseline function, and disease status. Reasons for offering a repeat craniotomy include obtaining a diagnosis when there is suspicion of malignant transformation, cytoreduction of progressive disease with a plan for adjuvant therapy postoperatively, obtaining tissue for clinical trial purposes, and debulking a large or symptomatic recurrence associated with mass effect. Patient should have a reasonable functional status and an expected survival longer than 1–3 months from surgery which is the expected recovery time from a craniotomy. As with first-time craniotomies, patients should be medically optimized before proceeding with surgery, and if there is a history of seizures, adequate seizure control with antiepileptic medications should be achieved prior to surgical intervention. Our group discusses patient management as part of a multidisciplinary team including neuro-oncologists, neurosurgeons, and radiation oncologists to determine if reoperation is the most reasonable avenue to pursue when a recurrence is detected.

### Surgical technique for repeat craniotomies

#### Skin opening

**Fig. 1:**
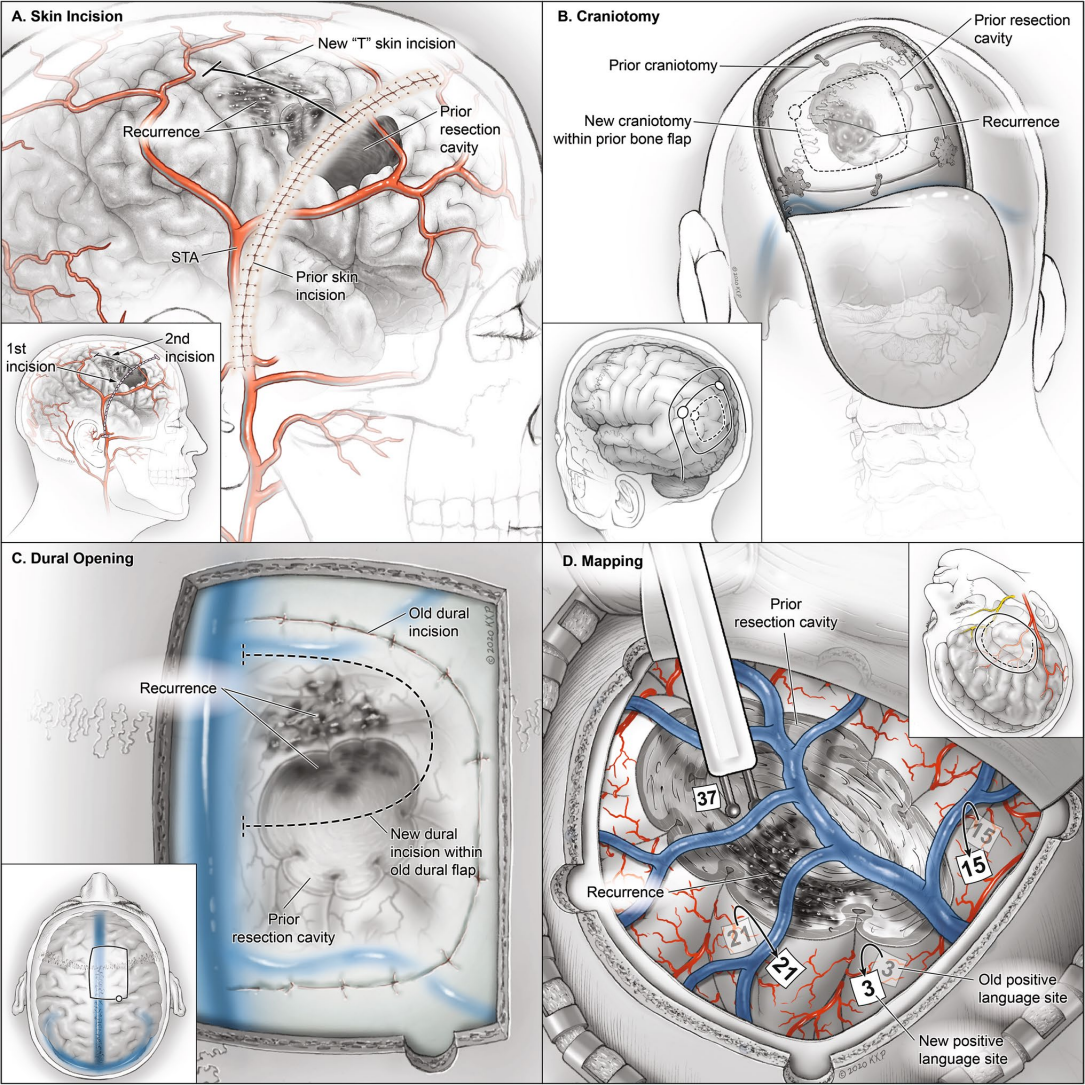
Technical considerations for approaching glioma recurrence. **a** The skin incision for a repeat craniotomy may not overly the area of tumor recurrence. If the exposure needs to be adjusted, then a perpendicular incision (a “T” incision) is used to extend the skin opening. **b** A bone flap that has not fused to the surrounding cranium may be removed and elevated. However, if the prior bone flap has fused to the calvarium, then the craniotomy can be tailored within the prior flap to expose the focus of recurrence. **c** The cortex tends to be most adherent to the dura under the prior suture line, and a new dural flap may be within the prior dural opening. If necessary, a leg of the prior suture can be crossed to obtain the necessary cortical exposure. **d** Mapping for glioma recurrence must be tailored to tumor location. A combination of cortical and subcortical mapping can be performed to allow for safe resection. Previously positive mapping sites at first surgery may not be positive at the time of repeat craniotomy given the ability of neighboring cortical regions to assimilate function

Assessing the integrity of the skin and identifying prior incisions is critical for any patient who is evaluated for surgery. Skin thickness, mobility, turgor, and presence of hair follicles can influence how the skin is opened, manipulated, and closed. After placing the patient in a Mayfield head-holder and registering neuro-navigation, the location of the tumor is drawn on the scalp to determine if the prior skin incision allows for sufficient access to the site of tumor recurrence. If the exposure needs to be adjusted, then a perpendicular incision (“T” incision) is used to extend the skin opening (Fig. 1a). Our group aims to keep the length of any skin flap shorter than its width to ensure adequate blood supply. The subcutaneous tissue is bluntly dissected beyond the skin incision to undermine the tissue and allow for a tension free closure. If the scalp is thin, Raney clips are avoided. Monopolar electrocautery is avoided during repeat craniotomies, and the bipolar is favored to control any arterial bleeding. After the bone exposure is complete, the galea is kept covered with a wet 4 × 4 gauze and soaked with betadine irrigation.

### Craniotomy

A craniotomy that facilitates targeted cortical mapping of the surrounding tissue and allows for a trajectory to the center of the tumor, termed the transcortical equatorial approach, is performed [30]. The tumor is again drawn on the bone to visualize the necessary craniotomy for exposure. If the bone flap from the prior surgery is mobile and overlies the lesion, then the plates and screws are removed, and the prior bone flap is elevated. If the prior bone flap has fused to the calvarium, then the craniotomy can be tailored within the prior flap to expose the focus of recurrence (Fig. 1b). In these cases, the dura may be adherent and prone to tearing during opening.

#### Dural opening

Dural opening during repeat craniotomies can be challenging. Once again, the lesion is drawn on the dura, and efforts should be made to stay within the prior suture line (Fig. 1c) as the cortex tends to be most adherent to the dura under the prior suture line. If necessary, a leg of the prior suture line can be crossed to obtain the necessary cortical exposure to perform a tailored map. To minimize infection, gloves are changed prior to opening the dura. During opening, the surgeon must be cautious to avoid avulsing cortical or bridging veins adherent to the ventral surface of the dura. In some recurrent cases, the dura is opened with a 15-blade and then the back of the blade is used to mobilize the underlying cortex away from the adherent dura with the assistance of copious irrigation. In general, scissors are avoided under the dura during recurrent tumor resections. Any bleeding dural vessels are snapped together with a hemostat to obtain hemostasis, and the bipolar is avoided to prevent dural shrinkage. Notably during mapping procedures, sites of cortical function may have migrated when compared to prior surgical function (Fig. 1d).

#### Closure

Dural closure can be difficult in the recurrence setting given the extent of dural scarring and friability. We aim to close the dura either primarily or with a dural patch in anticipation of a potential future surgery for tumor recurrence. The dural closure acts as a barrier between the cortical surface and bone and is critical to safely perform any future craniotomy. If the dura is hyperemic, we prefer to resect it and sew in a patch rather than coagulating the edges and shrinking it back. A dural sealant is applied, but it does not replace achieving a water-tight closure. If a large entry was made into the ventricular system near the foramen of Monro, a cavity drain is left in place for 2–3 days to divert bloody CSF. However, for smaller entries into the temporal or occipital horn, a drain is not required. The bone flap is replaced in the standard fashion with plates and screws, and vancomycin powder is applied to the wound [26]. A subgaleal drain is left in place if the subcutaneous tissue is vascular and bloody.

In the reoperation setting, skin closure poses some significant challenges. Prior radiation may lead to fragile skin which can make closure difficult. For a second craniotomy with healthy skin and no prior radiation, closure proceeds in the usual fashion with interrupted subgaleal sutures and skin staples to evert the edges. For any patient undergoing a third craniotomy or beyond with or without radiation, the skin is closed with a running vertical mattress 4–0 nylon. Sutures are kept in place for at least 10 days but may be left in up to 3–4 weeks if Bevacizumab was recently used given the concern for higher rates of surgical site infection (SSI).

### Identification of complications

Complication events were identified via chart review by the authors (R.A.M., J.S.Y., A.F.H., V.S.) and were categorized as either surgical or medical and were retrospectively collected within different timeframes in the postoperative setting: by discharge, between discharge and 30 days postoperatively, and between 30 and 90 days postoperatively. New neurologic deficits were noted if present by 90 days postoperatively. Reoperations were noted if they were related to a postoperative complication.

### Systematic analysis

A systematic review was performed in accordance with PRISMA guidelines (Supplemental Fig. 1). To identify articles eligible for analysis, a database search was performed using PubMed and SCOPUS. The following search terms were used: (“Glioma” OR “Glioblastoma”) AND (“Craniotomy” OR “Surgery”) AND (“Complications” OR “Safety”). Abstracts were screened for relevance (*n* = 987) with the following exclusion criteria: pediatric glioma, brachytherapy craniotomy outcomes, minimally invasive approaches, adjunct medication outcomes, studies exclusively evaluating infratentorial tumors, studies reporting a mix of tumor types without sufficient data for glioma outcome extraction, and insufficient data to extract outcomes of interest. The remaining studies were evaluated in full-text review (*n* = 148). Outcomes of interest included overall complication rate as well as specific complications such as intracranial hemorrhage, SSI/wound dehiscence, cerebrospinal fluid (CSF) leak, and venous thromboembolism (VTE). Papers with the necessary data to determine these outcomes were eligible for meta-analyses (*n* = 30; References [1, 2, 4, 6–11, 13, 14, 16–19, 21, 22, 24, 27, 32–35, 37, 39, 40, 42, 43, 45]).

### Statistical analyses

Descriptive statistics were used to define the patient cohort, treatment details, and clinical outcomes. An ANOVA was used to compare OR time and length-of-stay across number of prior craniotomies. A Pearson ${\chi }^{2}$ test was used to compare nominal variables, and a t-test was used to compare continuous variables across subgroups. A univariate nominal regression was performed to identify factors associated with postoperative complications by 90 days. Recursive partitioning analysis was used to identify the optimal split for white blood cell count (WBC) associate with complication outcome. This identified a WBC threshold of ≥ 21.7 as a cut off significantly associated with a postoperative complication. All patients with a WBC ≥ 21.7 were on high-dose steroids at the time of operation and 90% of these patients had been on chronic steroids for ≥ 1 month. Predictive variables from the univariate analysis with a *p*-value ≤ 0.2 were included in a multivariate nominal regression analysis. Meta-analyses for complication endpoints were performed using a random effects model. Heterogeneity was assessed using the ${\chi }^{2}$ test for heterogeneity, with *I*^2^ values ≥ 50% signifying sizable heterogeneity and ≥ 75% indicating substantial heterogeneity. Forest plots were used to present results. Statistical analysis was performed using JMP Pro 15 (SAS Institute, Cary, NC) and R software version 3.6.1 (R Foundation for Statistical Computing, Vienna, Austria). The level of significance was 0.05 for all analyses.

## Results

### Surgical cohort and complication profile

Over a 7-year period, 714 patients underwent a craniotomy for resection of either a newly diagnosed or recurrent glioma (WHO grade II–IV). All patients had 90-day follow-up to assess for postoperative complications and new neurologic deficits. Details of the cohort are found in Table 1. Within the cohort, 218 patients (30.5%) had undergone 1 prior craniotomy and 96 patients (13.5%) had undergone ≥ 2 prior craniotomies. Prior chemotherapy had been used in 5.8%, 50.9%, and 77.1% of patients undergoing 0, 1, or ≥ 2 prior craniotomies, respectively. Prior radiation had been used in 4.3%, 40.8%, and 55.2% of patients undergoing 0, 1, or ≥ 2 prior craniotomies, respectively.

**Table 1:** Patient demographics and treatment details

	All patients (*n* = 714)	First craniotomy (*n* = 400)	Repeat craniotomy (*n* = 314)	*p*-value
WHO grade				< .0001
II	248 (34.7%)	164 (41.0%)	84 (26.8%)	
III	206 (28.9%)	89 (22.3%)	117 (37.3%)	
IV	260 (36.4%)	147 (36.7%)	113 (35.9%)	
Age (mean ± STE)	47.3 ± 0.54	47.5 ± 0.78	47.0 ± 0.71	0.64
Sex (M:F)	422:292 (59.1%:40.9%)	247:153 (61.8%:38.2%)	175:139 (55.7%:44.3%)	0.10
Race/ethnicity *				0.12
African American	8 (1.1%)	6 (1.5%)	2 (0.6%)	
Asian/Pacific Islander	46 (6.6%)	30 (7.7%)	16 (5.1%)	
Caucasian	575 (80.5%)	306 (78.9%)	269 (86.5%)	
Hispanic/Latino	31 (4.4%)	20 (5.2%)	11 (3.5%)	
Other	39 (5.6%)	26 (6.7%)	13 (4.2%)	
ASA class				< .0001
I	42 (5.9%)	35 (8.8%)	7 (2.2%)	
II	405 (56.7%)	236 (59%)	169 (53.8%)	
III	263 (36.8%)	128 (32%)	135 (43.0%)	
IV	4 (5.6%)	1 (0.2%)	3 (1.0%)	
BMI (mean ± STE)	26.8 ± 0.2	26.9 ± 0.3	26.8 ± 0.3	0.86
Past medical history				
DM	35 (4.9%)	27 (6.8%)	8 (2.5%)	0.008
CHF	0 (0%)	0 (0%)	0 (0%)	NR**
HTN	116 (16.2%)	67 (16.8%)	49 (15.6%)	0.68
COPD	1 (0.1%)	1 (0.2%)	0 (0%)	0.28
Active smoker	34 (4.8%)	16 (4%)	18 (5.7%)	0.28
Number of prior craniotomies				
0	400 (56.0%)	400 (100%)	-	
1	218 (30.5%)	-	218 (69.4%)	
2	73 (10.2%)	-	73 (23.3%)	
3	21 (2.9%)	-	21 (6.7%)	
4	1 (0.2%)	-	1 (0.3%)	
5	1 (0.2%)	-	1 (0.3%)	
Prior chemotherapy	208 (29.1%)	23 (5.8%)	185 (58.9%)	< .0001
Prior radiation	159 (22.3%)	17 (4.3%)	142 (45.2%)	< .0001
Elective surgery Transfer/emergent case	680 (95.2%) 34 (4.8%)	374 (93.5%) 26 (6.5%)	306 (97.5%) 8 (2.5%)	0.01
Awake craniotomy Asleep craniotomy	358 (50.1%) 356 (49.9%)	231 (57.8%) 169 (42.3%)	127 (40.4%) 187 (59.6%)	< .0001
Skin closure				< .0001
Staples only	619 (86.7%)	370 (92.5%)	249 (79.3%)	
Suture only	67 (9.4%)	20 (5%)	47 (15.0%)	
Staples + suture	28 (3.9%)	10 (2.5%)	18 (5.7%)	
Drain placed	637 (89.2%)	394 (98.5%)	243 (77.4%)	< .0001

*DM*, diabetes mellitus; *CHF*, congestive heart failure; *HTN*, hypertension; *COPD*, chronic obstructive pulmonary disease; *OR*, operative room; *EBL*, estimated blood loss; *NR*, not reportable

^*^Declined to report race/ethnicity (*n* = 15 patients (2.1%))

^**^Not reportable given no patients in the cohort had CHF in either subgroup

The 90-day mortality rate for the cohort was 0%. Overall, there were 27 surgical and 10 medical complication events in 30 patients (4.2% of the cohort). First-time craniotomies were associated with 9 medical and 14 surgical complications seen in 4.3% of this subgroup while repeat craniotomies were associated with 1 medical and 13 surgical complications seen in 4.1% of this subgroup. New neurologic deficits were seen in 13.4% of patients by 90 days (first-time vs repeat craniotomy: 12.7% vs 14.0%, *p* = 0.62). Table 2 details the complication profile in patients undergoing a first-time craniotomy, and Table 3 details the complication profile in patients undergoing a repeat craniotomy. Overall, there were 19 reoperation events for complications in 19 patients (2.7% of the entire cohort). First-time craniotomies had a reoperation rate of 3.5% while repeat craniotomies had a reoperation rate of 2% (*p* = 0.22). Prior to discharge, 3 patients required reoperation for hematoma evacuation, decompression for stroke, and removal of a retained drain. Between discharge and 30 days postoperatively, there were 4 patients who required reoperation, all relating to wound infection or dehiscence. Between 30 and 90 days postoperatively, there were 12 return to operating room events: 7 for infection washout, 2 for hydrocephalus shunting, 2 for burr holes for subdural collections, and 1 for shunting of a persistent pseudomeningocele. Of all patients who developed a postoperative infection, only 1 patient received Bevacizumab which had been given in the postoperative period about 3 weeks after surgery.

**Table 2:** Overview of complication events for patients undergoing first craniotomy

	By discharge	Discharge to 30 days	30 days to 90 days
Return to OR events	2	2	4
Surgical complication events	2	9	3
EDH	0	0	0
SDH	0	2	0
IPH	1	2	0
Stroke	1	0	0
CSF Leak	0	0	0
SSI	0	3	2
Wound dehiscence	0	0	0
Subdural hygroma	0	2	1
Hydrocephalus	0	0	0
Medical complication events	3	6	0
Cardiac arrest/MI	0	0	0
PNA	1	3	0
VTE	1	3	0
Sepsis	0	0	0
UTI	0	0	0
AKI	1	-	-

*EDH*, epidural hematoma; *SDH*, subdural hematoma; *IPH*, intraparenchymal hematoma; *CSF*, cerebrospinal fluid; *SSI*, surgical site infection; *MI*, myocardial infarction; *PNA*, pneumonia, *VTE*, venous thromboembolism; *UTI*, urinary tract infection; *AKI*, acute kidney injury

**Table 3:** Overview of complication events for patients undergoing repeat craniotomy

	By discharge	Discharge to 30 day	30 days to 90 days
Return to OR events	1	2	8
Surgical complication events	1	5	7
EDH	0	0	0
SDH	0	0	1
IPH	0	0	0
Stroke	1	0	0
CSF leak	0	0	0
SSI	0	3	3
Wound dehiscence	0	0	1
Subdural hygroma	0	1	0
Hydrocephalus	0	1	2
Medical complication events	0	1	0
Cardiac arrest/MI	0	0	0
PNA	0	0	0
VTE	0	1	0
Sepsis	0	0	0
UTI	0	0	0
AKI	0	-	-

### Predictors of complications

Next, we examined if the number of prior craniotomies impacted treatment outcomes and complications (Table 4). Within this cohort, patients with prior craniotomies had shorter operation times (*p* < 0.0001) and lower estimated blood loss (EBL) (≥ 2 vs. 0 prior craniotomies, *p* = 0.03; 1 vs. 0 prior craniotomies, *p* = 0.0001) compared to patients undergoing initial craniotomy. The length-of-stay and home discharge rate were not statistically different between groups. Rates of patients suffering a complication or requiring a reoperation by discharge, 30 days, 90 days, or at any timepoint did not differ based on number of prior craniotomies.

**Table 4:** Number of prior craniotomies does not impact frequency of complications at discharge, 30 days, or 90 days

		Prior craniotomies			
		0	1	≥ 2	*p*-value
OR time (hrs)		6.9 ± 0.1	5.8 ± 0.1	5.4 ± 0.2	< .0001*
EBL (mL)		178.4 ± 6.1	139.2 ± 8.3	148.9 ± 12.3	0.0004*
Length of stay (d)		3.6 ± 0.14	3.6 ± 0.19	2.9 ± 0.29	0.11*
Discharge home		362/400 (90.5%)	201/218 (92.2%)	88/96 (91.7%)	0.76†
Patients with complication	By discharge	4/400 (1.0%)	1/218 (0.5%)	0/96 (0%)	0.50†
	Discharge to 30 days	13/400 (3.3%)	4/218 (1.8%)	2/96 (2.1%)	0.54†
	30 days to 90 days	3/400 (0.8%)	5/218 (2.3%)	2/96 (2.1%)	0.25†
	Surgery to 90 days	17/400 (4.3%)	9/218 (4.1%)	4/96 (4.2%)	0.99†
Patients requiring reoperation by 90 days ‡		8/400 (2%)	8/218 (3.7%)	3/96 (3.1%)	0.45†
Patients with new neurologic deficit by 90 days		56/400 (14%)	28/218 (12.8%)	12/96 (12.5%)	0.88
90-day mortality		0/400 (0%)	0/218 (0%)	0/96 (0%)	1.00

*OR*, operating room; *EBL*, estimated blood loss

^*^ANOVA

^†^*χ*^2^ test

^‡^Analysis by patient. One patient required with 1 prior craniotomy required 2 reoperations by 90 days

We next performed analyses to determine patient, tumor, and treatment factors that were predictive of postoperative complications (Table 5). On multivariate analysis, older age (unit OR by decade 1.5, 95%CI 1.0–2.2) and WBC ≥ 21.7 (OR 12.6, 95%CI 2.5–62.9) were significant predictors of postoperative complications. Given that SSI and wound dehiscence were the most frequent surgical complications observed, we examined patient risk factors that were associated with this complication specifically (Table 6). Higher preoperative BMI (*p* = 0.0003) and urgent/emergent surgery (*p* = 0.04) were associated with SSI/dehiscence. Risk factors predicting intracranial hemorrhage including epidural, subdural, or intraparenchymal hematomas were also assessed. A history of DM was associated with intracranial hemorrhage (DM vs no DM: 5.7% vs. 0.4%, *p* = 0.0003).

**Table 5:** Univariate and multivariate analysis for predictors of any complication

	Univariate analysis			Multivariate analysis		
	OR	95% CI	*p*-value	OR	95% CI	*p*-value
Age (by decade)	1.4	1.1–1.8	0.01	1.5	1.0–2.2	0.04
BMI	1.1	1.0–1.1	0.03	4.5	0.25–82.1	0.31
Grade			0.14			0.98
IV vs II	2.0	0.8–4.7		1.1	0.3–3.6	
IV vs III	2.2	0.8–5.7		1.0	0.3–3.2	
Sex (M:F)	1.0	0.5–2.2	0.92			
Prior craniotomies			0.99			
1 vs 0	1.0	0.4–2.2				
≥ 2 vs 0	1.0	0.3–2.9				
Race *			0.19			0.59
African American	4.0	0.5–33.8		6.0	0.6–61.9	
Asian/Pacific Islander	1.3	0.3–5.6		1.6	0.3–8.1	
Hispanic/Latino	1.9	0.4–8.6		1.8	0.3–11.8	
Other	3.2	1.0–9.8		2.3	0.5–11.3	
ASA classification			0.06			0.53
II vs I	0.6	0.1–2.8		0.3	0.1–1.9	
III vs I	1.2	0.3–5.5		0.5	0.1–2.9	
IV vs I	6.7	0.5–96.4		1.1	0.03–48.2	
Diabetes	3.2	1.1–9.9	0.03	1.5	0.4–5.8	0.53
Active smoker	0.7	0.1–5.1	0.71			
Hypertension	2.7	1.2–6.0	0.01	1.7	0.6–4.7	0.30
Prior chemo	1.0	0.5–2.3	0.91			
Prior XRT	1.1	0.4–2.5	0.89			
Transfer/emergent (vs elective)	1.5	0.3–6.4	0.62			
Drain	1.7	0.4–7.4	0.46			
Skin closure			0.44			
Suture	0.6	0.2–2.8				
Staples + suture	7.1^e−7^	0-∞				
Awake craniotomy (vs asleep)	1.1	0.5–2.4	0.72			
OR time	1.0	0.1–13.6	0.99			
EBL (by 100 mL)	1.2	1.0–1.4	0.03	1.2	0.9–1.4	0.18
Preoperative WBC ≥ 21.7	11.4	2.8–46.6	< .0001	12.6	2.5–62.9	0.002

*BMI*, body mass index; *XRT*, radiation therapy; *OR*, operating room; *EBL*, estimated blood loss

^*^vs Caucasian

**Table 6:** Risk factors for surgical site infection or dehiscence

	SSI/dehiscence (*n* = 11)	No wound issues (*n* = 703)	*p*-value
Age	55.1 ± 4.3	47.2 ± 0.5	0.07*
BMI	32.6 ± 1.6	26.8 ± 0.2	0.0003*
Male Female	4/422 (0.9%) 7/292 (2.4%)	4/418 (99.1%) 285/292 (97.6%)	0.12†
Prior craniotomies			0.77†
0	5/400 (1.3%)	395/400 (98.7%)	
1	4/217 (1.8%)	213/217 (98.2%)	
2 +	2/97 (2.1%)	95/97 (97.9%)	
ASA classification			0.13†
I	2/42 (4.8%)	40/42 (95.2%)	
II	3/405 (0.7%)	402/405 (99.3%)	
III	6/263 (2.3%)	257/263 (97.7%)	
IV	0/4 (0%)	4/4 (100%)	
Diabetes	0/35 (0%)	35/35 (100%)	0.45†
No diabetes	11/679 (1.6%)	668/679 (98.4%)	
Prior XRT	3/159 (1.9%)	156/159 (98.1%)	0.69†
No prior XRT	8/555 (1.4%)	547/555 (98.6%)	
Elective	9/680 (1.3%)	671/680 (98.7%)	0.04†
Transfer/emergent	2/34 (5.9%)	32/34 (94.1%)	
Drain	10/637 (1.6%)	627/637 (98.4%)	0.86†
No drain	1/77 (1.3%)	76/77 (98.7%)	
Skin closure			0.50†
Staples only	9/619 (1.5%)	610/619 (98.5%)	
Suture only	2/67 (3.0%)	65/67 (97.0%)	
Staples + suture	0/28 (0%)	28/28 (100%)	

*BMI*, body mass index; *SSI*, surgical site infection; *XRT*, radiation therapy

^*^t-test

^†^*χ*^2^ test

### Meta-analyses of complications after glioma resection

**Fig. 2:**
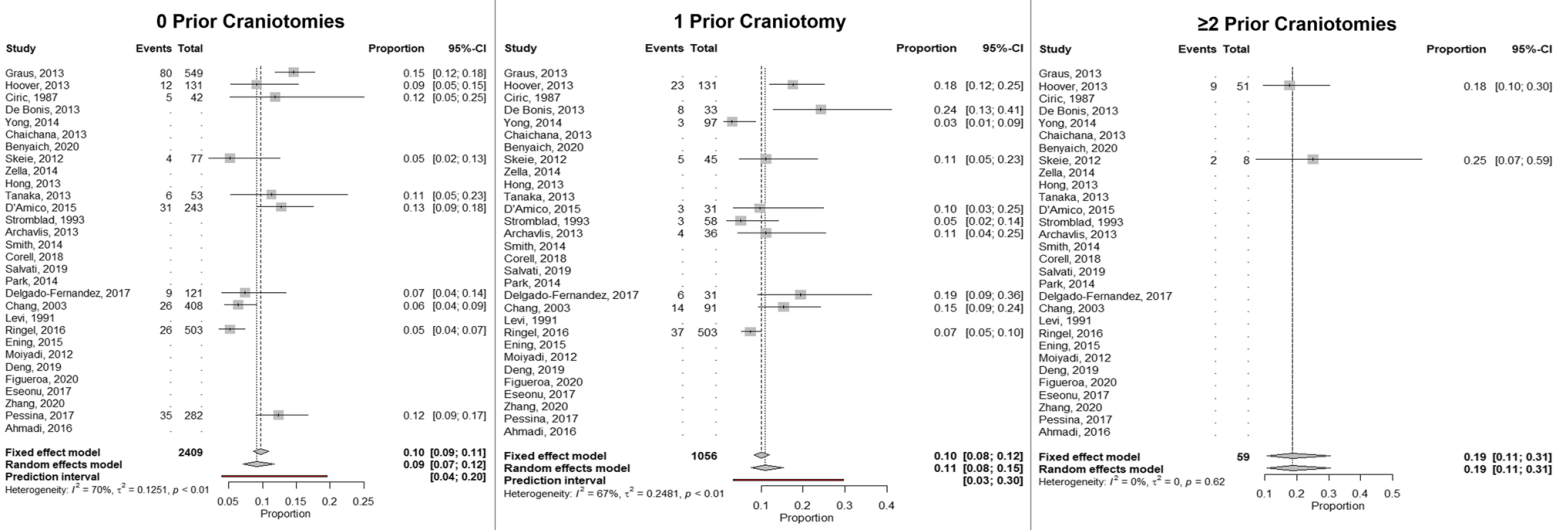
Forest plot analysis of overall complication rate by number of prior craniotomies

A systematic analysis of studies examining glioma patients undergoing either initial or repeat craniotomy for resection was performed, and 30 studies met criteria for analysis. Overall, 10 studies examined complications after first-time craniotomy, 10 studies examined complications after 1 prior craniotomy, and 2 studies examined complications after 2 or more prior craniotomies. Overall non-deficit complication rates after 0, 1, and ≥ 2 prior craniotomies were 9%, 11%, and 19%, respectively, based on a random effects model (Fig. 2).

**Fig. 3:**
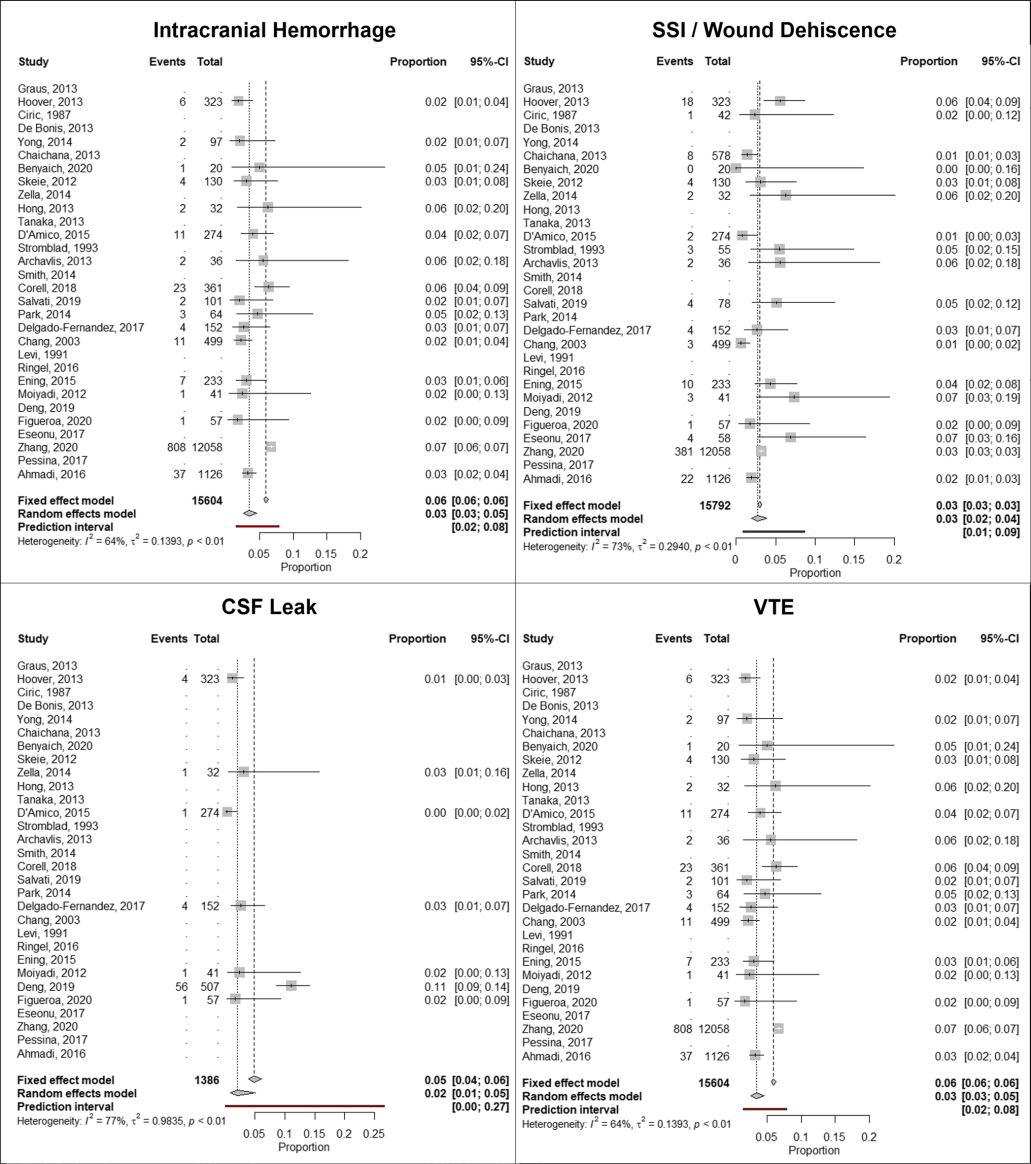
Forest plot analysis of specific complications including intracranial hemorrhage, SSI/wound dehiscence, CSF leak, and venous thromboembolism (VTE)

Forest plots of rates of specific complications are displayed in Fig. 3. In the 17 studies that examined rates of intracranial hemorrhage after craniotomy for glioma, the proportion of cases with intracranial hemorrhage was 3%. In the 7 studies that examined rates of CSF leak, the proportion of cases with CSF leak was reported to be 2%. In the 18 studies that examined rates of SSI or wound dehiscence, the proportion of cases was reported to be 3%. In the 13 studies that examined VTE rates, the proportion of cases was reported to be 3%.

## Discussion

In this series of glioma patients undergoing craniotomy for resection, we retrospectively evaluated the complication profile under the care of an experienced neurosurgeon specializing in glioma surgery and examined whether number of prior craniotomies impacted complications and reoperations. This modern series of patients in the era of electronic medical records ensured more comprehensive complication inclusion as well as provided for better delineation on when these complications occurred during a patient’s postoperative course. Overall, total complication rates by 90 days in patients who had undergone 0, 1, or ≥ 2 prior craniotomies were 4.3%, 4.1%, and 4.2%, respectively. Furthermore, rates of new neurologic deficits by 90 days did not differ between first-time and repeat craniotomies. In addition to results from the meta-analysis examining glioma-specific complication rates, the rate of morbidity in this series is also lower than other large center modern series examining complication rates for intracranial tumors [44]. In this series, repeat operations tended to have shorter operating room times and less EBL compared to primary operations. This likely reflects the goal of these operations to remove focal recurrence through small, tailored exposures. Reoperation rates for complications were also low in this series and did not differ by number of prior craniotomies. Given these findings, repeat craniotomies should therefore be considered when patients re-present with focal recurrent disease. Furthermore, it is critical that complication rates be kept low so as to not detract from the survival benefit that more extensive tumor resection can achieve in the context of recurrent glioma [33, 41].

When examining risk factors for complications in the entire cohort, age and significant leukocytosis were both found to be predictors of overall complications. Age has previously been identified as a risk factor for poor prognosis in the glioma population [23, 28]. Significantly elevated WBC seen in a subgroup of patients on high-dose steroids may predispose patients to higher risks of complications. Despite prior concern regarding radiotherapy, diabetes, and reopening of a prior incision, these were not identified as risk factors for an SSI. Interestingly, diabetes was identified as a risk factor for intracranial hemorrhage, which may be related to microvascular disease associated with this disease context and warrants further investigation.

Surgeon case volume has previously been associated with improved patient outcomes in a variety of neurosurgical disease contexts [12, 36]. Within this single-surgeon cohort, rates of complications were similar between initial and repeat craniotomies suggesting that consistent implementation of “best-practice” techniques can help achieve low complications rates and allow repeat craniotomies to be safe. The surgical techniques described above detail our practice and experience with strategies to minimize complications during reoperations. However, other surgeons may have alternative methods that they have found to minimize complications. When a complication event occurs, the triggering issues must be identified, and adjustments in practice must be made to avoid similar subsequent events. This process requires that neurosurgeons monitor their own complications over the course of their own careers. By doing so, techniques and management decision may be tailored to promote better patient outcomes.

There are several limitations to this study. Complications were assessed retrospectively based on documentation within the electronic medical record. Inherent to all systematic meta-analyses, there may be reporting and publication bias present. Furthermore, we could not control for case difficulty across studies, tumor biology and patient comorbidities and variations in adjuvant treatment practices. Being a single-surgeon series may limit the generalizability of these results. However, this single surgeon’s experience also limited variability in practice, with the goal of describing “best practice” techniques that could be used to achieve low surgical and medical operative morbidity when resecting recurrent glioma.

## Conclusions

In this single-surgeon series, the complication profile in patients undergoing repeat craniotomy for gliomas was similar to patients undergoing primary resection and can be kept to a low level. The development of best practices over one’s career can help mitigate complications and allow resection of recurrent glioma through a repeat craniotomy to be safely performed.

## Supplementary Information

Below is the link to the electronic supplementary material.

Supplementary Fig. 1 (PNG 527 KB)

High Resolution Image (TIF 242 KB)

## Data Availability

May provide as supplemental data if accepted.
