# Supplementary figures and images for: Reducing complication rates for repeat craniotomies in glioma patients: a single-surgeon experience and comparison with the literature
Source: Acta Neurochir (Wien). 2021 Dec 30;164(2):405–17. doi: 10.1007/s00701-021-05067-9 (PMC8854329; doi:10.1007/s00701-021-05067-9)

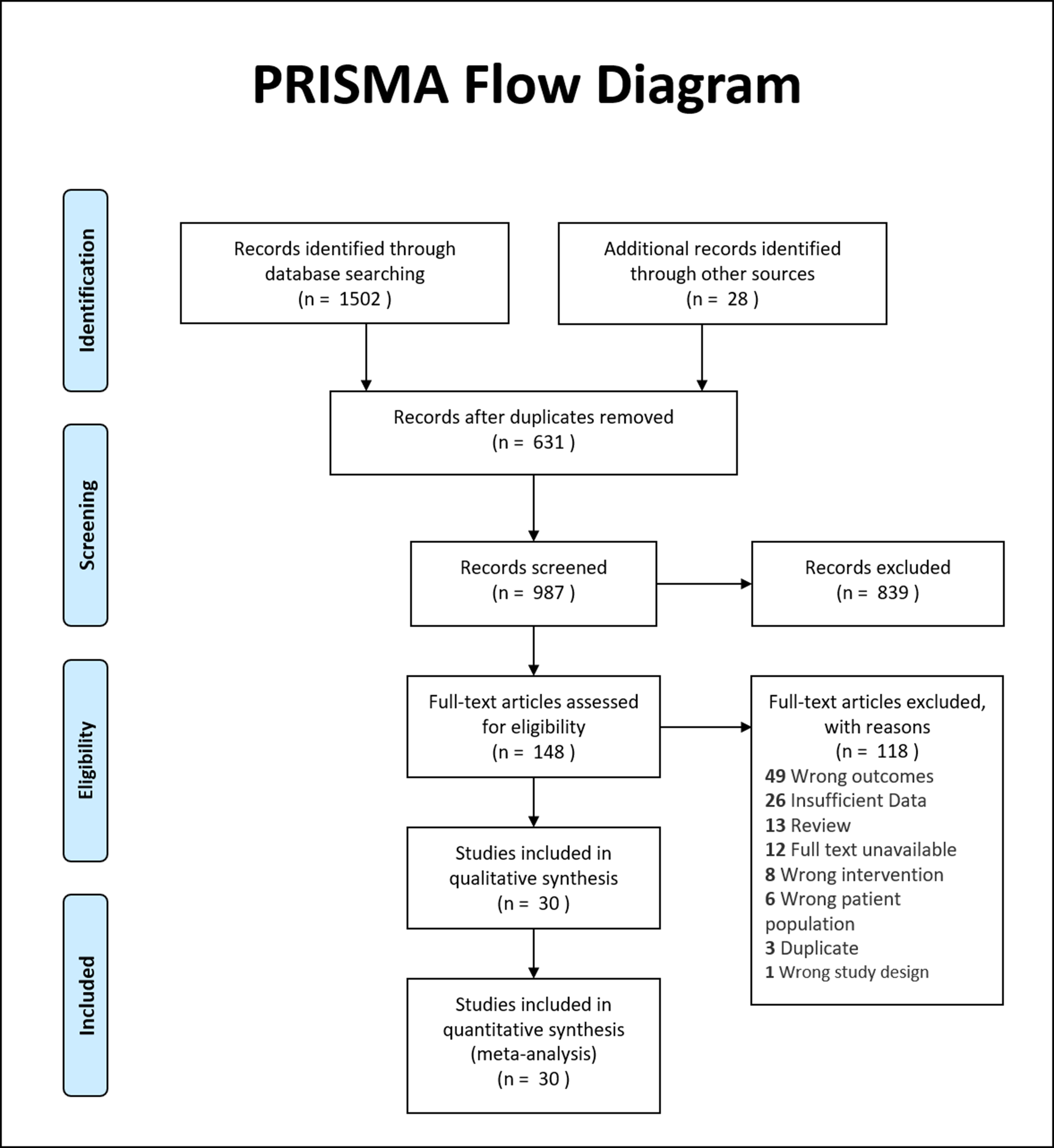

Supplement: Supplementary file 1 — (PNG 527 KB) [file 701_2021_5067_Fig4_ESM.png]

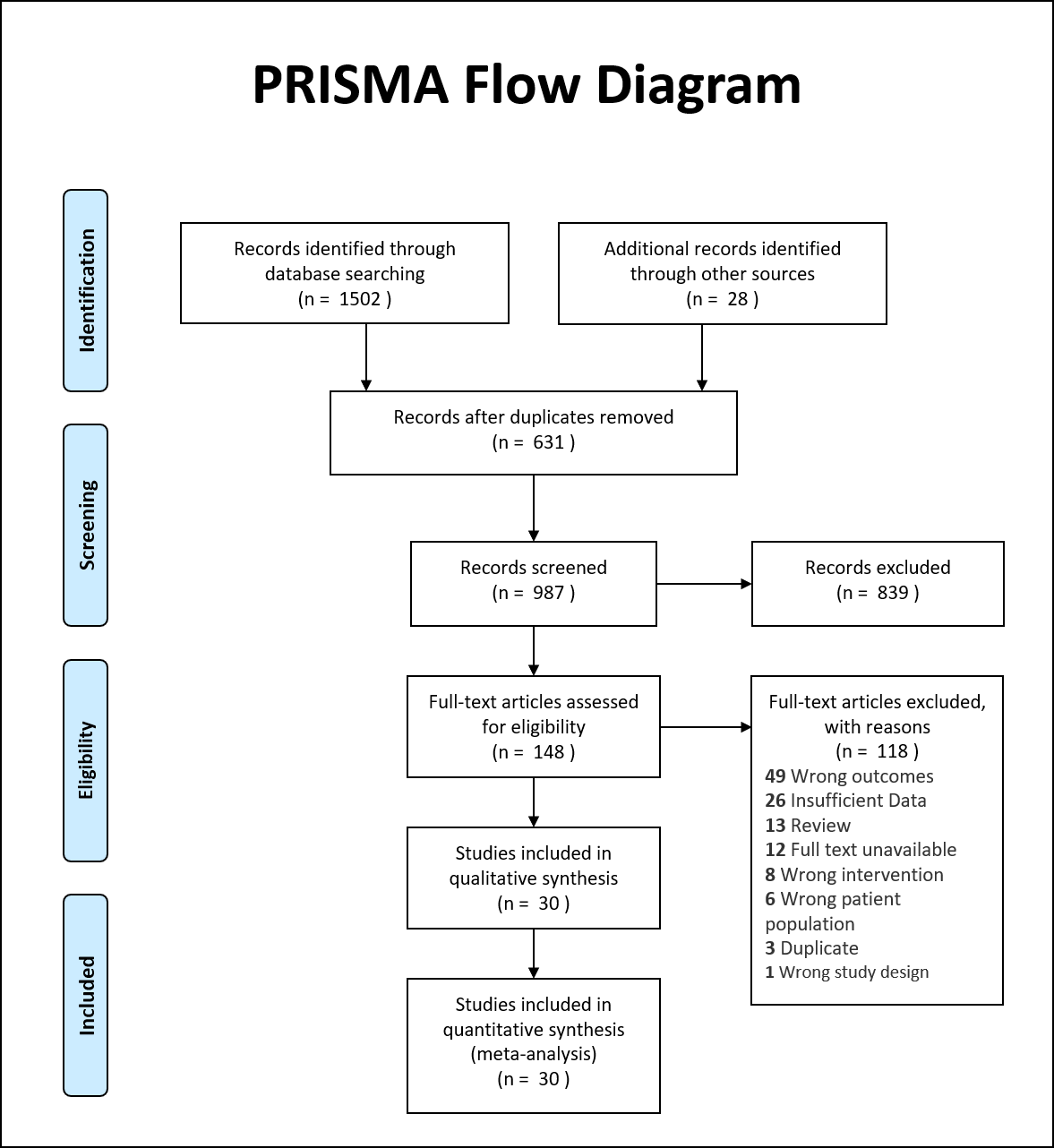

Supplement: Supplementary file 2 — High Resolution Image (TIF 242 KB) [file 701_2021_5067_MOESM1_ESM.tif]
